# Translational treatment of aphasia combining neuromodulation and behavioral intervention for lexical retrieval: implications from a single case study

**DOI:** 10.3389/fnhum.2015.00447

**Published:** 2015-08-19

**Authors:** Elizabeth E. Galletta, Amy Vogel-Eyny

**Affiliations:** ^1^Speech-Language Pathology and Audiology, Hunter CollegeNew York, NY, USA; ^2^Speech-Language-Hearing Sciences, Graduate Center, City University of New YorkNew York, NY, USA

**Keywords:** aphasia, neuromodulation, treatment, lexical retrieval, tDCS

## Abstract

**Background:** Transcranial direct current stimulation (tDCS), a non-invasive method of brain stimulation, is an adjunctive research-therapy for aphasia. The concept supporting translational application of tDCS is that brain plasticity, facilitated by language intervention, can be enhanced by non-invasive brain stimulation. This study combined tDCS with an ecologically focused behavioral approach that involved training nouns and verbs in sentences.

**Method:** Participant: A 43-year-old, right-handed male with fluent-anomic aphasia who sustained a single-left-hemisphere-temporal-parietal stroke was recruited.

Treatment: Instrumentation included the Soterix Medical 1 × 1 Device. Anodal tDCS was applied over Broca’s area. Behavioral materials included: sentence production, naming in the sentence context, and implementation of a social-conversational-discourse treatment.

Design and Procedures: The independent variable of this crossover case-study was tDCS, and the dependent variables were language and quality-of-life measures. In each session the subject received language treatment with the first 20 minutes additionally including tDCS.

**Results:** Performance in naming nouns and verbs in single words and sentences were obtained. Verb production in the sentence context increased after active anodal tDCS and speech-language treatment.

**Conclusion:** Aphasia treatment that involves naming in the sentence context in conjunction with translational application of tDCS may be a promising approach for language-recovery post stroke.

## Introduction

Aphasia is a cognitive language disorder that is manifested in language comprehension and production deficits. It affects stroke survivors worldwide, and although research has focused on behavioral treatments for language recovery (see review, Brady et al., 2012), communication deficits often persist. Many studies indicate that individuals with aphasia experience lexical retrieval deficits for both nouns (Howard et al., 1985; Nettleton and Lesser, 1991; Doesborgh et al., 2004) and verbs (Marshall et al., 1998; Carragher et al., 2013; Takizawa et al., 2015), and cognitive neuropsychological models have been proposed to explain lexical impairments for spoken words (e.g., Dell et al., 1997; Levelt, 1999). Related to these models, several forms of behavioral therapy have been developed and utilized over the years to address word-naming difficulties in individuals with aphasia. Some of these interventions are categorized as semantic treatments and some are phonological treatments.

Semantic treatment tasks may include a variety of activities such as auditory word-to-picture matching, written word-to-picture matching, and yes/no verification tasks. Also, within this type of treatment, semantic feature description tasks may be implemented that include distinguishing features among similar objects, and classifying semantic features into categories. Other semantic treatments can include activities such as implementing a specific matrix that includes categorization, function, attribute, or association tasks, with all of these activities relating a semantic cue to the target.

Phonological treatments are different from semantic treatment approaches and target breakdown at a different level within the general frame of the cognitive neuropsychological model approach to intervention. Examples of phonological treatment tasks include rhyming, syllable-number verification, oral-word reading, word repetition, and phonological cueing hierarchy (e.g., the clinician first presents a rhyming word, then an initial phoneme cue, and finally a repetition cue). Not infrequently, both semantic and phonological treatments are utilized to target retrieval of nouns at the single-word level (e.g., Howard et al., 1985; Nettleton and Lesser, 1991; Doesborgh et al., 2004); however, in recent years treatment has been extended to approaches that train naming at the sentence level (Raymer and Kohen, 2006; Conroy et al., 2009).

Behavioral verb treatments have also largely been targeted at the single-word level (Mitchum and Berndt, 1994; Marshall et al., 1998; Raymer and Ellsworth, 2002; Wambaugh et al., 2002, 2004; Rodriguez et al., 2006; Rose and Sussmilch, 2008; Boo and Rose, 2011; Carragher et al., 2013) and have been approached from a semantic and a phonological perspective. Sentence-level treatments for verb production have focused on treating verbs as single words in a sentence context (Bastiaanse et al., 2006; Edwards and Tucker, 2006; Raymer and Kohen, 2006; Links et al., 2010; McCann and Doleman, 2011) and on treating verb argument structure or syntax (Thompson et al., 1997, 2013; Helm-Estabrooks and Nicholas, 2000; Kim and Thompson, 2000; Schneider and Thompson, 2003; Webster et al., 2005; Kim et al., 2007; Wambaugh and Ferguson, 2007; Edmonds et al., 2009; Webster and Gordon, 2009). While training verb production at the single-word level in isolation or a sentence context may focus more directly on semantics or phonological cueing, training syntax may also indirectly promote lexical retrieval of both nouns and verbs, since production of a syntactically appropriate utterance includes a verb, and almost always requires a noun. Moreover, often semantic and phonological cues are embedded within sentence training programs (e.g., frequently the sentence context provides a semantic cue, and within a training program a model for repetition is provided, which is a phonological cue), even when the focus is on training at the syntactic level of production.

Although behavioral therapy studies of aphasia have resulted in some support for word retrieval gains, communication impairments often remain after behavioral treatment. Indeed, even when there are observed improvements in retrieval for treated items, generalization to untreated items following intervention is uncommon (see reviews: Webster and Whitworth, 2012; Best et al., 2013), though some evidence suggests that training complex verbs generalizes to the production of untrained, less complex verbs (e.g., Thompson et al., 2013). It seems that training sentences may be a method that promotes generalization more consistently than training single words alone.

In response to the noted challenges for promoting generalization and language recovery for aphasia, in recent years, a new technique of therapeutic importance known as transcranial direct current stimulation (tDCS), a non-invasive form of brain stimulation, has increasingly been utilized to modulate language and cognitive abilities. While the application of tDCS as a non-invasive therapeutic tool used to induce changes in neural excitability (Datta et al., 2009; Salvador et al., 2010) is gaining interest, the specific cellular targets of stimulation remain unclear. During tDCS, current flow (1–2 mA; milliamps) from an anode to a cathode electrode generates weak electrical fields (EFs) across the cortex (EFs <1 V *m*^-1^). Since tDCS has been found to modulate cortical activity in the motor cortex (Nitsche and Paulus, 2000, 2001; Antal et al., 2004), this method has recently been applied translationally to the cortical areas subserving language. Early studies outside the motor cortex had the initial focus of using this technique to consider language modulation in healthy populations, largely as proof-of-concept research (e.g., Iyer et al., 2005; Ross et al., 2011; Meinzer et al., 2014). While many studies report a simple excitation and inhibition relationship between the anode and the cathode electrodes, the exact nature of the neuronal mechanism that induces increased or decreased neuronal firing and how that translates to behavioral outcome measures is unknown (Datta et al., 2009). In spite of the underspecified neural mechanisms underlying potential language gains, research conducted in healthy populations using tDCS is being applied translationally to clinical populations after initial animal studies demonstrated this treatment is safe to administer (for a review of tDCS used in animal studies see Brunoni et al., 2011).

Research in the application of tDCS to clinical populations has largely included individuals who experience declines in language and/or cognitive functions including patients with Parkinson’s disease (Pereira et al., 2013), Schizophrenia (Schretlen et al., 2014), and Alzheimer’s disease (Ferrucci et al., 2008), with promising results suggesting improvements in language skills when combining tDCS with a behavioral therapy protocol. The translational application of tDCS techniques from early proof-of-concept studies in the healthy literature (e.g., Iyer et al., 2005; Flöel et al., 2008; Sparing et al., 2008) to aphasic populations, as a way of potentially improving upon the gains made in language abilities following behavioral treatment alone, began in 2008 when the first study that implemented tDCS with a group of chronic, non-fluent stroke survivors (Monti et al., 2008), was published. This initial study did not include a behavioral treatment component; nonetheless, it informs aphasia researchers and motivates current protocols, which generally involve the application of tDCS as an adjuvant to promote neuroplasticity when combined with behavioral treatment for language recovery post stroke.

Although still a relatively new area of research, an increasing number of studies utilizing tDCS techniques in aphasic language research have examined lexical retrieval abilities, with a predominant focus on noun retrieval (e.g., Fiori et al., 2011, 2013; Flöel et al., 2011; Kang et al., 2011). The behavioral component of these studies has generally approached anomia treatment through single-word, picture-naming tasks involving common nouns, and this form of therapy has been applied during both online and oﬄine tDCS. As well, several different tDCS montages (i.e., the placement of electrodes on the scalp) have been employed in conjunction with behavioral treatment in aphasic populations to investigate noun-retrieval performance following stimulation with mixed results. For example, anodal stimulation of 1 mA over Wernicke’s area (denoted as location CP5 using the 10–20 EEG configuration system) in a group of non-fluent aphasic subjects improved accuracy and reaction time in a picture-naming task, and gains were maintained 1 and 3 weeks post-stimulation (Fiori et al., 2011). In addition, a second study using this montage and this approach found improved naming accuracy (response time was not assessed) in a different group of non-fluent aphasics (Fiori et al., 2013), suggesting this paradigm may be promising. Similarly, configurations stimulating over Broca’s area have observed improvement in noun retrieval following 1 mA of anodal-tDCS (a-tDCS; Costa et al., 2015); however, other research employing 2 mA of anodal stimulation has not found such an effect (Volpato et al., 2013), signifying that increasing the level of the current is not necessarily better for improvement of behavioral outcomes. Interestingly, administering 2 mA of cathodal tDCS (c-tDCS) over the right Broca’s area homologue was found to improve the retrieval of nouns compared to sham stimulation (Kang et al., 2011).

While the montages differ among studies, the theory of aphasia that focuses on inter-hemispheric competition motivates the approaches taken by all of these researchers (Galletta et al., 2015a,c; Shah-Basak et al., 2015). In general the motivation in applying a-tDCS to the left hemisphere is to target preserved peri-lesional cortex (e.g., Fridriksson et al., 2011) while the application of cathodal stimulation to the right hemisphere homologue is implemented to down-regulate an overactive right hemisphere (e.g., Kang et al., 2011; also see review by Schlaug et al., 2011). Yet in reality, this somewhat simple interpretation of the cellular effects of tDCS is misleading because both a-tDCS and c-tDCS can either excite or inhibit neuronal firing depending upon a variety of neuronal factors (Datta et al., 2009). Taken together, then, neither the animal studies that consider tDCS at the cellular level nor the behavioral studies reported here definitively specify which approach is the ideal montage and configuration for the improvement of lexical retrieval in individuals with aphasia (Galletta et al., 2015b). Recent research looking at inter-individual variability in language performance outcomes following differing montages (Shah-Basak et al., 2015) suggests that individuals with aphasia may show unique responsiveness to montage configurations depending on lesion location, size, and individual differences among subjects. In fact the wide variety of montages (e.g., left hemisphere anodal, right hemisphere cathodal, etc.) cited as improving lexical retrieval suggests that there may not be one best montage that promotes language recovery post stroke. Rather, the optimal montage for the promotion of language recovery may be best specified on a case-by-case basis.

While initial tDCS aphasia studies focus on lexical retrieval of nouns, more recently, researchers have investigated the effect of tDCS on the verb retrieval abilities of individuals with aphasia (Fiori et al., 2013; Marangolo et al., 2013, 2014; Volpato et al., 2013; Costa et al., 2015; Manenti et al., 2015). As with the noun retrieval literature, studies examining verb recovery often employ confrontation-naming paradigms with mixed results. Volpato et al. (2013) have found no effect of 2 mA of a-tDCS over left Broca’s area (intersection of T3–Fz and F7–Cz based on the 10–20 EEG system) on noun or verb retrieval. However, other studies utilizing a frontal configuration have found a benefit. That is, Costa et al. (2015) suggest that bihemispheric tDCS (note that while Costa et al. use the term “bihemispheric tDCS” Nasseri et al., 2015 suggest a framework that prefers the term “bilateral bipolar-balanced tDCS”) over Broca’s area (F5 – anodal) and its homologue (F6 – cathodal) at 1 mA leads to improved performance on noun and verb retrieval compared to sham tDCS. Researchers have also examined the effect of a-tDCS over the left dorsolateral prefrontal cortex (DLPFC) in a single-case study while simultaneously applying c-tDCS over the right hemisphere homologue and have found greater verb naming compared to baseline at 48 weeks post treatment (Manenti et al., 2015).

Although this research suggests that there are improvements in noun and verb retrieval following behavioral treatment at the single-word level coupled with tDCS over left frontal regions, such as Broca’s area, gains are often small, despite being significant, and often recovery is limited to the treated items (see Monti et al., 2013 for a review). Only one study that directly trained verb naming (Manenti et al., 2015) has reported generalization to untrained items, and none of the studies that directly trained lexical retrieval of nouns have noted generalization to untrained items. The question remains whether generalization to retrieval of an untrained set of items can be found when including an ecologically-valid behavioral therapy in conjunction with tDCS rather than a behavioral treatment that trains only at the single-word level, since in the real world people rarely speak in single words. That is, including naming at the sentence level may provide a functional and beneficial method for treating lexical retrieval, combined with tDCS.

This was a double-blind, cross-over, sham-controlled case study. Both the subject and the clinicians implementing the behavioral treatment were blind to the tDCS condition (sham tDCS or a-tDCS). Only the first author, who was administering the tDCS, knew whether the subject was receiving sham tDCS or a-tDCS. The purpose of this study was to investigate whether translational application of non-invasive brain stimulation in the form of tDCS, coupled with behavioral treatment that includes training naming in sentences, promotes language improvement in an individual with anomic aphasia. In previous tDCS aphasia studies, naming was mostly trained in the single-word context; no study to date has implemented an ecological approach that included training naming in the sentence context. We assessed the feasibility of this novel approach to behavioral treatment by way of a case study design. In this study, a-tDCS applied over Broca’s area, combined with an ecologically focused behavioral therapy that included naming in the sentence context, was administered to investigate if this approach improves noun and verb retrieval in an individual with fluent, anomic aphasia.

## Materials and Methods

### Participant

This study was approved by the Hunter College IRB, and the participant signed the Informed Consent. The individual with aphasia was a right-handed, 43-year-old male who had 16 years of education. He experienced a single left-hemisphere stroke 20 months before entering the study. A clinical MRI of the brain was performed without contrast at the time of the stroke. Axial diffusion-weighted, axial T2, and axial FLAIR sequences were performed. Findings indicated that a large wedge-shaped region of restricted diffusion involving the left parietal and temporal lobes was observed, compatible with ischemia. There was a mild associated local mass effect on regional sulci without midline shift or downward transtentorial herniation. The subject participated in speech-language intervention prior to this study but was not receiving any individual behavioral treatment outside of his participation in this research during the time period of this study.

The participant presented with fluent aphasia and was classified as having anomic aphasia according to the Western Aphasia Battery-Revised (WAB-R, Kertesz, 2006). A WAB-R aphasia quotient of 81 (range: 0–100) indicated a mild severity of aphasia yet the participant reported significant difficulty with naming both nouns and verbs. See WAB-R profile in **Table 1**.

**Table 1:** Sociodemographic and clinical features.

*Gender*	*M*
*Age*	43
*Education level*	16
*Time post onset*	1 year and 8 months
*Western Aphasia Battery-Revised (WAB-R)*	
Spontaneous speech	18/20
Auditory verbal comprehension	172/200
Repetition	74/100
Naming and word finding	69/100
Aphasia quotient	81.8/100
*Phonemic fluency*	
“F” production	0
“A” production	2
“S” production	5

The participant was a monolingual speaker of English and had normal or corrected-to-normal vision and hearing was within normal limits. The participant had no history of a neurological illness other than a single left hemisphere temporal parietal ischemic stroke and no psychiatric illness. He met the tDCS exclusionary criteria, which included no history of seizures or epilepsy, and no metal implants anywhere in the body.

### Stimuli Selection

Given the participant’s self-reported difficulty with nouns and verbs, speech therapy targeted noun and verb production in a sentence context.

#### Assessment Materials

**FIGURE 1:**
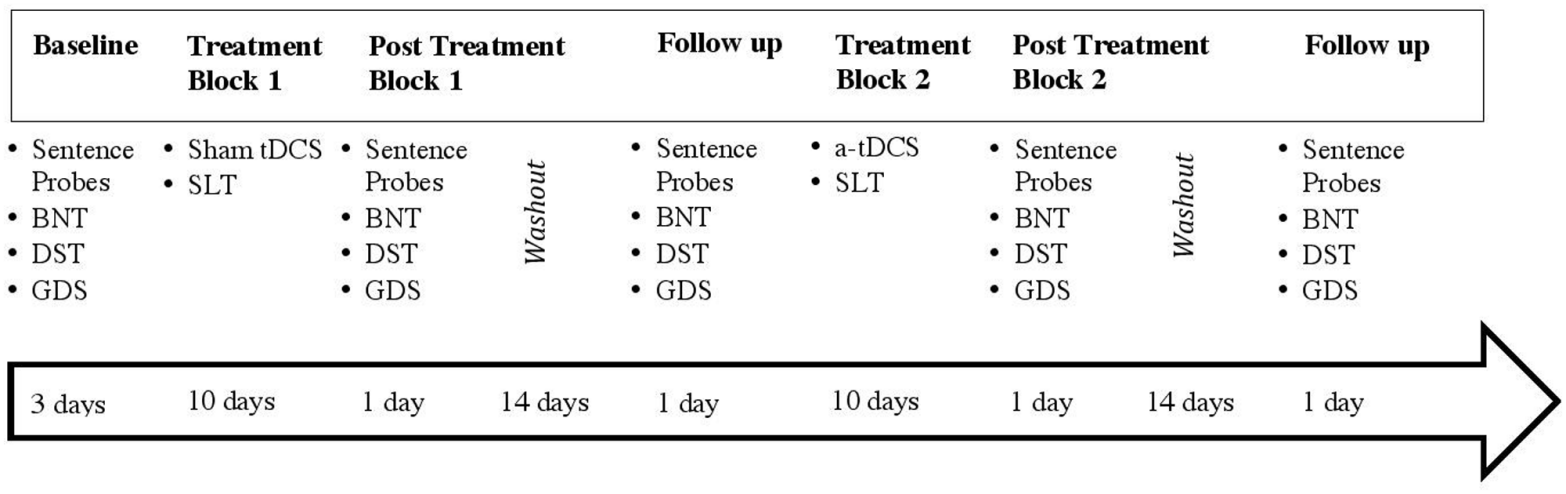
**Timeline and experimental design of the study**.

The participant was administered a set of probe tasks to assess word retrieval at five testing points during the study on an untrained set of sentence-embedded nouns and verbs (see **Figure 1**) that consisted of a series of colored pictures that represent transitive verbs in a sentence context (e.g., “*He makes the bed*”). Sentences for these probes were taken from a study that examined noun and verb retrieval using transitive action pictures (Raymer and Kohen, 2006). Modification of the sentences from Raymer and Kohen (2006) included removal of sentences that contained repeat nouns or verbs so that no sentence included an overlapping target noun or verb with another sentence. Additional probe sentences were also created [additional to Raymer and Kohen’s (2006) list of items, though adhering to the same syntactic structure] in order to create several lists of probes with no overlapping nouns or verbs on any of the lists. The sentences were divided into seven lists of 10 sentences, with three lists given in the first phase of the study (pre-treatment baseline), and the remaining four lists were administered across four separate sessions (post treatment block 1, post treatment block 1 follow-up, post treatment block 2, and post treatment block 2 follow-up). See assessment timeline, **Figure 1**. Lists were matched for frequency of nouns (Kucera and Francis, 1967), and name agreement for target nouns and verbs depicted in the action pictures was established by polling four independent raters; name agreement was 100% for all items. The participant was presented with each action picture on a computer screen, and the administrator asked, “What is happening?” All responses were recorded online and later transcribed. The correct percentage of noun and verb productions was calculated for each of the seven probe lists.

An additional set of outcome measures was also administered. Performance on the Boston Naming Test (BNT; Kaplan et al., 1983), a 60-item picture-naming task, was assessed. There was one administration with 60 items in the first phase, pre-treatment baseline, and two administrations of the 60-item BNT post-treatment (blocks 1 and 2). As well, assessments were given in order to determine if gains made following tDCS were attributable to non-specific effects, such as enhanced physiological arousal. These included the Driving Scenes Test (DST) of the Neuropsychological Assessment Battery (NAB; Stern and White, 2003), which is a measure of visual attention. In the DST, the participant viewed a colored picture from the perspective of a driver behind the wheel of a car. He was then shown a different picture and was asked to point out or state anything that was different between the two driving scenes. There were five scenes on the DST and these five scenes were administered in the first phase, pre-treatment baseline; the DST was administered two times post-treatment (blocks 1 and 2). As well, the Geriatric Depression Scale (GDS; Yesavage et al., 1982–1983) is a paper and pencil questionnaire that is widely used as a self-reporting tool of depression among adults. There were 30 items on the GDS and these 30 items were administered in the baseline pre-treatment phase; there were two administrations of the GDS post-treatment.

The experimental design of the study (see **Figure 1**) shows that the participant underwent two treatment blocks (sham tDCS followed by a-tDCS), each lasting 10 consecutive sessions with a 2-week washout period between blocks. Baseline measures were collected over the course of 3 days to assess pre-treatment word-naming abilities. Outcome measures including the BNT, DST, GDS, and untrained sentence probes were employed in post-treatment and follow-up sessions after each treatment block.

### Behavioral Treatment Materials

Speech therapy consisted of 60-minute sessions that included three components for 20 minutes each. The 60-minute treatment sessions are the conventional length for behavioral therapy, and the 20-minutes of tDCS reflects the literature that has used tDCS with behavioral therapies. The first treatment component was a modified sentence production protocol based on the Sentence Production Program for Aphasia (SPPA; Helm-Estabrooks and Nicholas, 2000). The behavioral activity of the modified SPPA was sentence production without direct focus on lexical retrieval. This was included as an alternate approach to training lexical retrieval in the sentence context, which was the second component of treatment that consisted of a modified sentence-embedded naming production protocol (Raymer and Kohen, 2006). The modified sentence-embedded naming treatment included training specific lexical items within sentences. The last component of therapy was a focused-discourse treatment activity, which involved conversational discourse. All three behavioral treatment activities involved training at the sentence or discourse levels; there was no lexical retrieval treatment at the single-word level. The three behavioral treatment activities are described in further detail in the sections that follow. A speech-language-pathologist (author Elizabeth E. Galletta) trained four graduate student clinicians at Hunter College, City University of New York, who administered the behavioral therapy protocol. These clinicians were blind to the tDCS condition. All sessions were video-recorded and reviewed by two independent graduate clinicians, who rescored productions for accuracy. In the sections that follow, a detailed description of the behavioral treatment procedures are described under three headings: Sentence Production Training, Sentence-Embedded Production Training (SEPT), and Focused Discourse.

#### Sentence Production Training

This treatment approach involved training several sentence types. Although the SPPA protocol (Helm-Estabrooks and Nicholas, 2000) comprises eight sentence types, for the purposes of this modified SPPA protocol for this single case study, four sentence types were chosen to be used for training sentence production. These sentence types included: declarative transitive (e.g., “*I teach school”*), declarative intransitive (e.g., “*The girl dances*”), imperative transitive (e.g., “*Pour the wine*”), and imperative intransitive (e.g., *“Wake up”*). The Helm-Estabrooks and Nicholas (2000) criterion for progressing from one sentence type to the next was followed (13/15 correct). Based on the progression guidelines of the SPPA Administration Manual, the participant trained only on the first sentence type (i.e., declarative transitive) during this portion of the treatment since the participant did not meet the criterion to advance, even though four sentence types were planned as potential targets for training during treatment.

Administration of this treatment program involved scaffolding and prompting. The participant viewed a black and white picture of the to-be-produced sentence, and the administrator modeled the sentence within a larger narrative framework that acted as a carrier phrase. Here is an example of an item within a larger narrative context: “*When people ask Ginny what she does for a living, she says, ‘I teach school”’* (Helm-Estabrooks and Nicholas, 2000). The first prompt required the participant to repeat the target sentence and provided the participant with a carrier sentence in order to facilitate production of the target sentence: “*When people ask Ginny what she does for a living, she says, ‘I teach school.’ What does Ginny say?”* If the participant achieved the correct production after this first probe, a more difficult, second-level probe that did not include the target sentence was given: “*When people ask Ginny what she does for a living, what does she say?”* Scoring of participant productions was consistent with the guidelines provided in the SPPA Administration Manual. A response was considered fully correct and received one point if the target sentence was either produced in its entirety, self-corrected successfully, produced along with other meaningful words, or a grammatical response was provided with the same syntax as the target sentence but with a semantically related word substituted. A partially correct response received half a point if one word was omitted or incorrectly produced, and a response was marked as incorrect and received no points if two or more words were omitted or produced incorrectly. If the participant did not produce the sentence following the first probe, then the administrator moved on to the next sentence rather than the second-level probe. If the participant did not produce the sentence following the second-level probe, the administrator moved on to the next sentence as well. Advancement to the next sentence-type was planned when the participant met the criterion of 13/15 correct productions after the second-level probe (criterion taken from Helm-Estabrooks and Nicholas, 2000). However, only the declarative transitive sentence type was trained since the subject did not meet the criterion to move to an additional sentence type.

#### Sentence-Embedded Production Training

A sentence-embedded production training protocol developed by Raymer and Kohen (2006), in which a sentence corresponding to a specific action picture is produced, was included in treatment in modified form. As in the modified SPPA, the same four sentence types were chosen to target noun and verb retrieval alongside the pictures provided in that treatment protocol. Through hierarchical and guided support from the clinician, the participant retrieved the target noun or verb in a sentence context. First, while the participant looked at a picture with the sentence beneath it, the administrator provided a verbal model of the sentence (e.g., the clinician said, “I teach school”). Next the participant read aloud the sentence with semantic prompts (e.g., if the difficulty was in producing the verb *teach* the clinician would say, “the word is similar in meaning to instruct, what is another word for instruct?”) and phonological prompts (e.g., the word starts with a /t/ sound) from the clinician as needed. A production was considered successful at the second step when the participant read the sentence, with or without prompts, in its entirety. As a final step, the clinician covered the noun, verb, or complete sentence, and the participant then generated the entire sentence including any covered targets. The response was only considered successful when the participant produced the complete target sentence. After completing production of three trained sentences, a barrier activity was included in order to reinforce spontaneous production. The participant and clinician had the same three pictures in front of them during the barrier activity, and out of view of the clinician, the participant spontaneously produced a sentence corresponding to one of the pictures. The clinician then identified the picture based on the participant’s production. The same criterion (13/15 correct) as used for the SPPA for progressing through these sentence types was employed for SEPT treatment; the participant progressed through all four of the sentence types during the SEPT portion of the treatment protocol.

#### Focused Discourse

This treatment did not follow a strict protocol for advancing to a higher level as the entire 20 minutes of this part of the 60-minute session focused on a discussion about a topic of shared interest. The clinician brought a current newspaper to each session and the subject and clinician looked through the paper together and chose a news story or theme to discuss. Neither the subject nor the clinician read the newspaper article in advance, but rather, it served as a strategy to come up with a shared topic of interest for discussion. Typically, the clinician and participant flipped through the paper for 2–3 minutes and then chose a topic that interested them based on the story headlines and photos. Examples of topics included were current events, sports, and weather. After choosing a topic, the clinician used supportive conversational strategies such as following the speaker’s lead and rephrasing what the speaker stated to facilitate the focused conversation.

### Transcranial Direct Current Stimulation

Stimulation was delivered through a constant current with two 35 cm^2^ (5 cm × 7 cm) saline-soaked sponges (Soterix Medical 1 × 1 device). The anode was centered over Broca’s area (F7) according to the international 10–20 EEG system, which prior research has shown to be a valid method for determining skull correlates of cortical locations (e.g., Koessler et al., 2009; Kang et al., 2011). Recent research suggests that Broca’s area is involved in lexical retrieval and thereby is an appropriate neural site to target in individuals with aphasia during tDCS (e.g., Holland et al., 2011). The cathode electrode was placed over the contralateral supraorbital area. Both active and sham stimulation occurred during the first 20 minutes of the behavioral treatment hour. Active stimulation was ramped up to 1 mA within the first minute of the treatment. During sham tDCS, the current similarly increased to 1 mA within the first minute and then was ramped down slowly to 0 mA after 1 min, where it remained for the duration of the 20-minutes tDCS portion of the session. As this was a double-blind study, the participant and the clinician administering the lexical-retrieval therapy were naive to the type of stimulation received. The first author of the study administered tDCS (Elizabeth E. Galletta) out of view of the clinician who administered the behavioral treatment, and out of view of the participant. The participant’s subjective ratings of pain and discomfort were collected at the end of every tDCS session as a safety measure with a protocol in place if pain or discomfort were reported. In addition, at the end of each treatment block, the subject was asked whether he thought the sessions included a-tDCS or sham tDCS.

### Procedure

**FIGURE 2:**
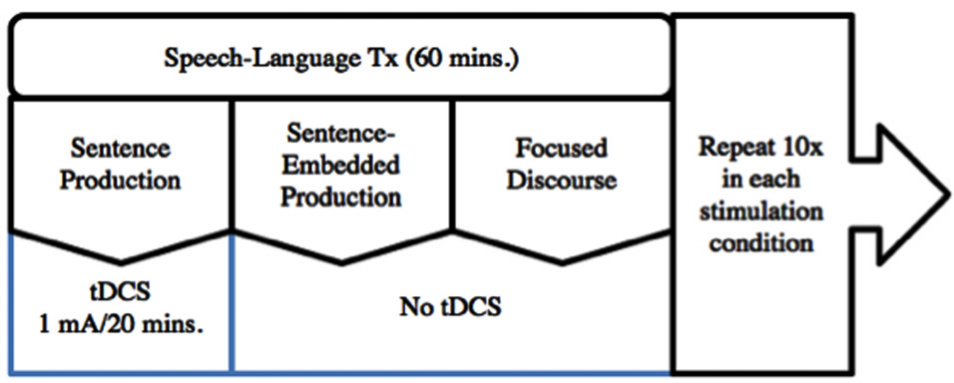
**One iteration of the experimental therapy protocol**.

There were two treatment blocks. The participant underwent 10 days of tDCS in conjunction with the behavioral intervention for each experimental block, a-tDCS and sham tDCS. Sham stimulation was delivered in the first block and a-tDCS in the second block. The behavioral intervention consisted of 20 minutes of each of the three treatment types, and for every session the order of the treatment was systematically alternated in a serial order (e.g., sentence treatment, sentence-embedded treatment, focused-discourse; sentence-embedded treatment, focused-discourse, sentence treatment; focused-discourse, sentence treatment, sentence-embedded treatment, etc.). The two conditions were separated by a 2-week washout period. Following application of the electrodes the tDCS device was turned on, and the participant received 20 minutes of stimulation in conjunction with one of the treatment interventions. The tDCS was then turned off and the behavioral intervention continued for the remainder of the hour (see **Figure 2**). Pre- and post-testing measurements consisted of the lists of sentence probes, the BNT, the GDS, and the DST. Pre-testing occurred before each treatment block and post-testing occurred immediately after each treatment block and again 2 weeks post each treatment block.

### Statistical Analyses

The Tau-U statistic (Parker et al., 2011) was used to compare the different intervention phases. Tau-U is a robust, non-parametric technique that has been shown to perform better than other non-parametric methods for analyzing single-case data (Brossart et al., 2014). Tau-U accounts for non-overlap data points between treatment phases, and it provides an effect size measure that indicates the proportion of change or improvement between phases. Following Ferguson (2009), Tau-U values between 0.2 and 0.5 are interpreted as small-to-moderate intervention effects and values between 0.5 and 0.8 as moderate-to-strong intervention effects. In addition, Tau-U can control for trends and autocorrelation effects within and between phases (Parker et al., 2011).

## Results

### Noun and Verb Performance

Descriptive and inferential statistics are reported in **Tables 2**–**4**, respectively. In **Tables 2** and **3** the “*n*” represents the stimulus items from the three phases of testing: collapsed baseline, post treatment block, and follow-up assessments. That is, in order to maximize the power of the study analyses, given that there were few stimulus items within a given task and testing time point, data within the three testing phases were not aggregated, and each single item (i.e., “*n*”) was the unit of analysis for both descriptive and inferential statistics (see **Table 2** for descriptive statistics by session): phase 1 baseline (composed of three baseline assessments), phase 2 post-sham (composed of post-treatment block 1 and follow-up to post-treatment block one assessments), and phase 3 post a-tDCS (composed of post-treatment block 2 and follow-up to post-treatment block two assessments). **Figure 1** displays the timeline for all phases of the study.

**Table 2:** Mean task accuracy by session.

	Noun probes		Verb probes		Geriatric Depression Scale (GDS)		Boston Naming Test (BNT)		Driving Scenes Test (DST)	
Session	*n*	Mean (SD)	*n*	Mean (SD)	*n*	Mean (SD)	*n*	Mean (SD)	*n*	Mean (SD)
Baseline 1	10	0.5 (0.53)	10	0.5 (0.53)	30	0.83 (0.38)	60	0.57 (0.5)	5	0.41 (0.1)
Baseline 2	10	0.6 (0.52)	10	0.8 (0.42)	-	-	-	-	-	-
Baseline 3	10	0.7 (0.48)	10	0.3 (0.48)	-	-	-	-	-	-
Post-Tx Session 1	10	0.5 (0.53)	10	0.3 (0.48)	30	0.9 (0.31)	60	0.58 (0.5)	5	0.48 (0.23)
Follow-up Session 1	10	0.5 (0.53)	10	0.7 (0.48)	30	0.87 (0.35)	60	0.65 (0.48)	5	0.62 (0.28)
Post-Tx Session 2	10	0.8 (0.42)	10	0.9 (0.32)	30	0.87 (0.35)	60	0.68 (0.47)	5	0.60 (0.13)
Follow-up Session 2	10	0.8 (0.42)	10	0.9 (0.32)	30	0.77 (0.43)	60	0.68 (0.47)	5	0.63 (0.14)

**Table 3:** Mean task accuracy at three phases of testing.

	Baseline			Post sham transcranial direct current stimulation (tDCS)			Post a-tDCS		
	*n*	Mean	SD	*n*	Mean	SD	*n*	Mean	SD
Noun probes	30	0.6	0.49	20	0.5	0.51	20	0.8	0.41
Verb probes	30	0.53	0.51	20	0.5	0.51	20	0.9	0.31
GDS	30	0.83	0.38	60	0.88	0.32	60	0.82	0.39
Boston Naming Test (BNT)	60	0.57	0.5	120	0.62	0.49	120	0.68	0.47
Driving Scenes Test (DST)	5	0.41	0.1	10	0.55	0.25	10	0.61	0.13

*n* = number of stimulus items.

**Table 4:** Inferential statistics (Tau-U).

	Baseline vs. Post sham tDCS				Post sham tDCS vs. Post a-tDCS			
	Tau-U	*Z*	*p*-value	90% CI	Tau-U	*Z*	*p*-value	90% CI
Noun Probes	-0.16	-0.6	0.545	-0.595< >0.275	0.48	1.81	0.069	0.045 < > 0.915
Verb Probes	-0.03	-0.113	0.91	-0.465< >0.405	0.56	2.117	0.034	0.125 < > 0.995
GDS	0.0167	0.111	0.912	-0.231< >0.264	-0.1	-0.658	0.511	-0.346 < > 0.148
BNT	0.0389	0.367	0.713	-0.135< >0.213	0.07	0.698	0.485	-0.1 < > 0.248
DST	0.48	1.253	0.21	-0.15< >1.11	0.28	0.731	0.465	-0.35 < > 0.91

**FIGURE 3:**
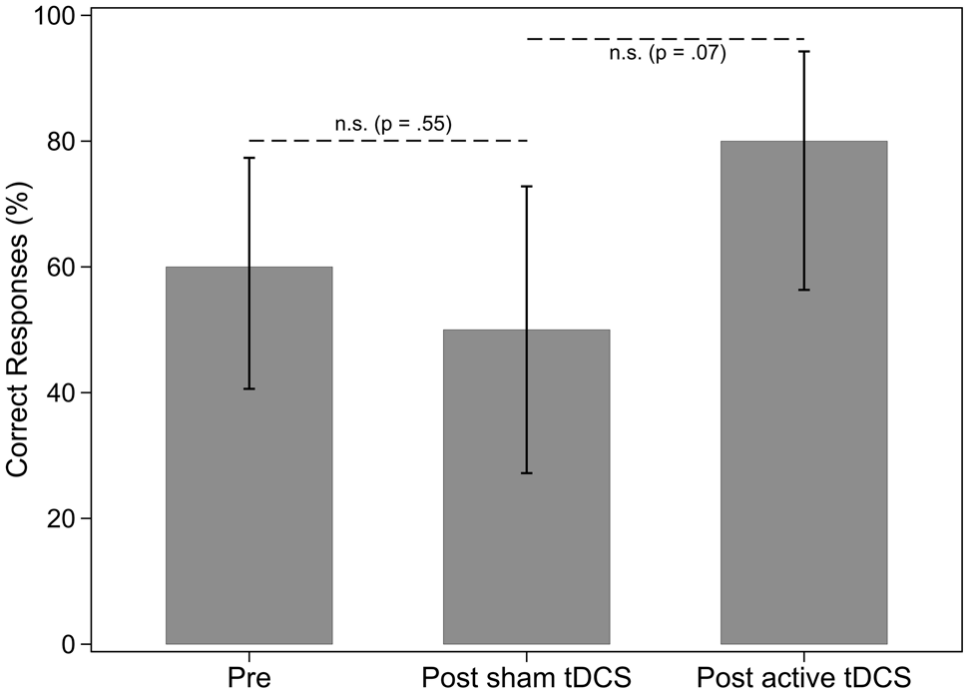
**Percentage of nouns in sentences correctly identified, by treatment phase**. Error bars: 95% CI.

**FIGURE 4:**
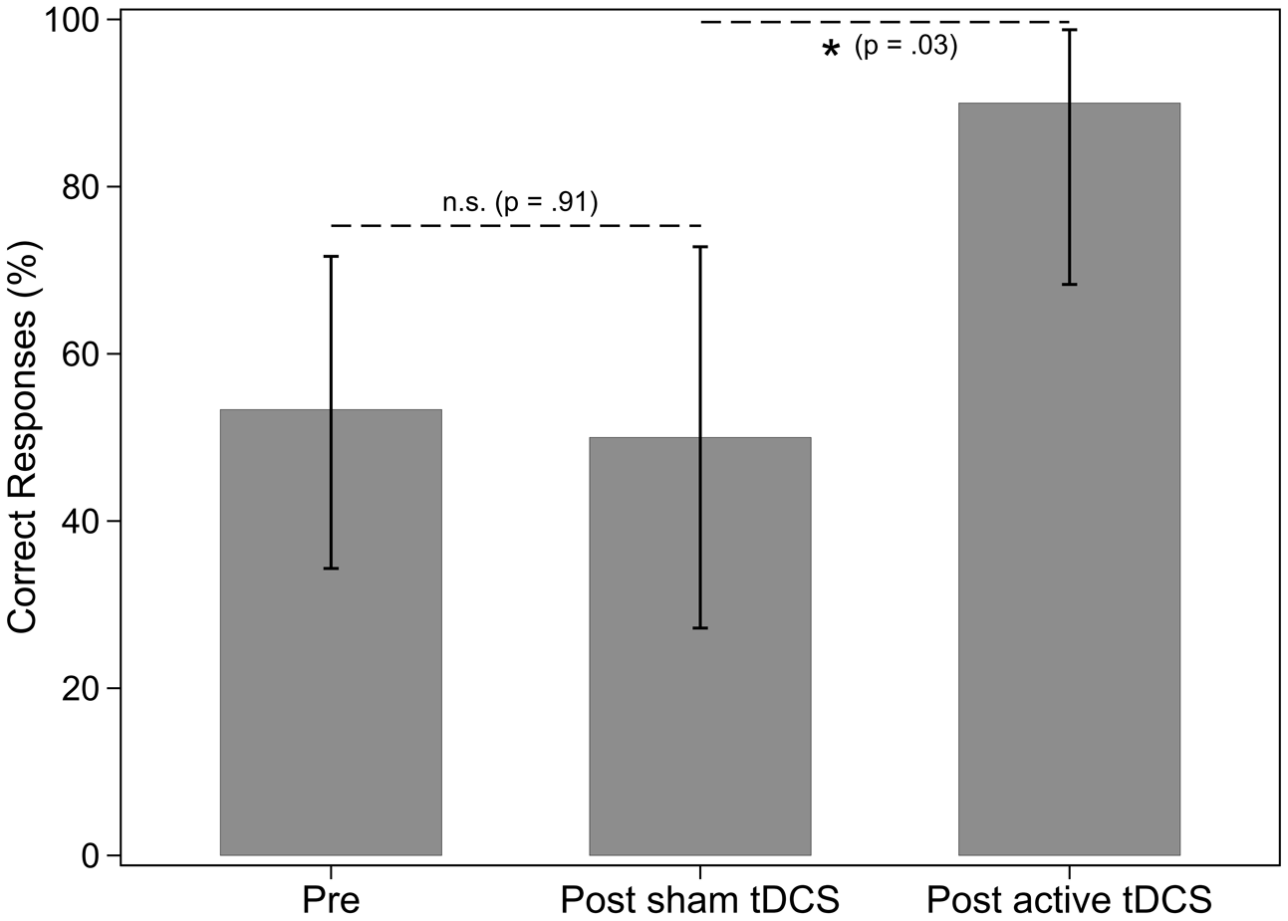
**Percentage of verbs in sentences correctly identified, by treatment phase**. Error bars: 95% CI.

**FIGURE 5:**
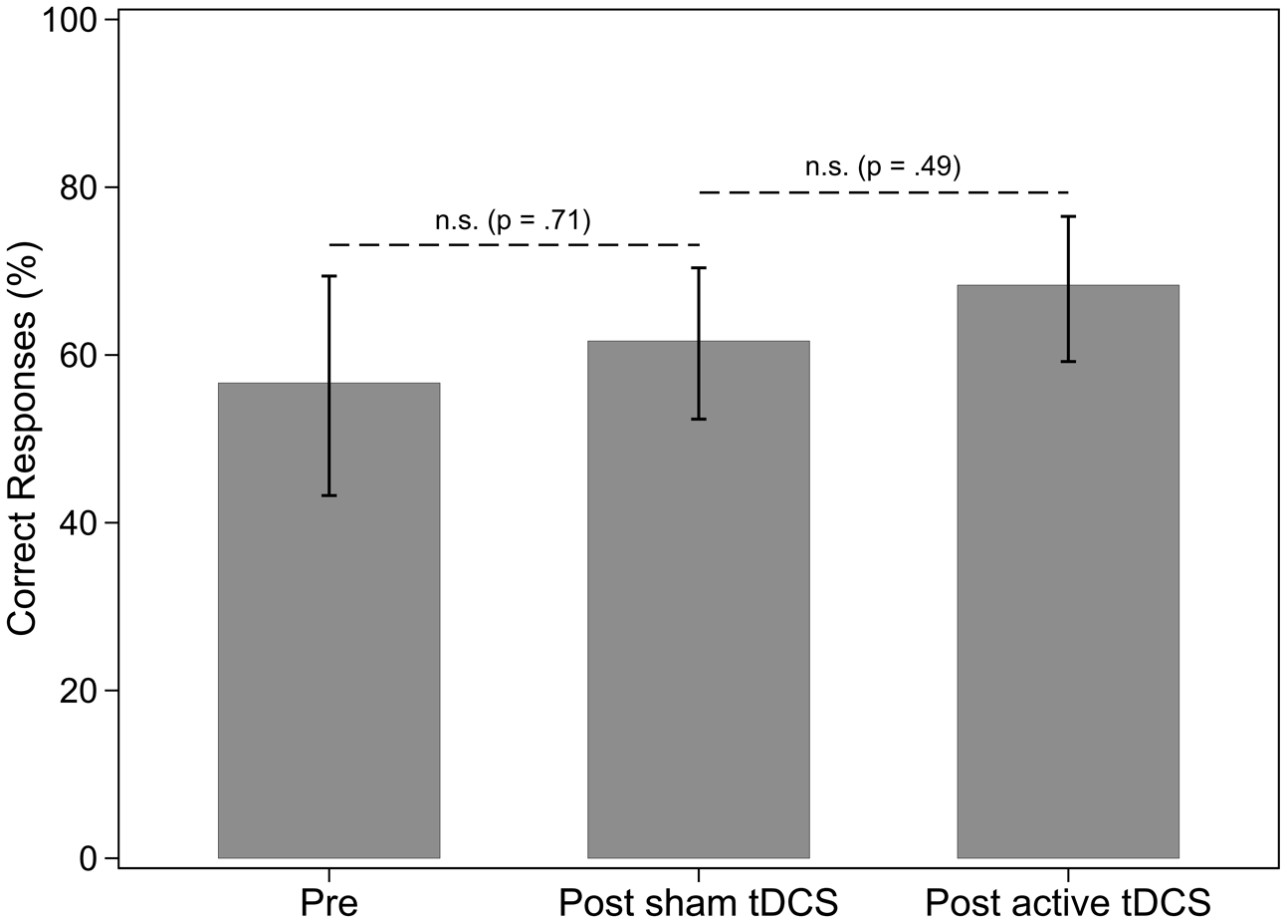
**Percentage of nouns in single words correctly identified, by treatment phase**. Error bars: 95% CI.

There were three baseline assessments in phase one for nouns and verbs in sentences. Results of the Tau-U analysis showed that the baseline percent correct for nouns and verbs did not differ [Tau-U = -0.12, *Z* = -0.45, *p* = 0.65, 90% CI (-0.555,0.315)]. Therefore the participant did not appear to have a deficit in one grammatical class over the other at baseline. Regarding percentage of correct noun responses when nouns were named in sentences, there was no effect of sham tDCS and no effect of a-tDCS for nouns (see **Table 4** and **Figure 3**). There was no effect of sham tDCS for retrieval of verbs in sentences (see **Figure 4**). However, there was a significant difference between post sham tDCS and post a-tDCS for retrieval of verbs in sentences (**Figure 4**). We take note that there was a statistically not significant 3% decrease in performance after sham tDCS and a statistically significant 56% improvement (a moderate-to-strong intervention effect) in performance after a-tDCS for verbs. Analysis of single-word retrieval for nouns as measured by the BNT indicated that there was no effect of sham tDCS on single word retrieval in isolation following sham tDCS or a-tDCS (see **Table 4** and **Figure 5**).

### Attention and Mood

There was no effect of sham tDCS or a-tDCS on attention as measured by the DST (see **Table 4**) or mood as measured by the GDS (see **Table 4**).

## Discussion

In this study, tDCS, a non-invasive brain stimulation technique, was applied in combination with an ecologically focused behavioral treatment approach. This sham-controlled, double-blind, cross-over study implemented a functionally relevant behavioral intervention that included sentence production, naming in the sentence context, and focused-discourse tasks in conjunction with tDCS in order to examine if treatment of nouns and verbs in a sentence context generalizes to an untrained set of sentence-embedded nouns and verbs in an individual with fluent anomic aphasia. Our results show that there was a moderate-to-strong effect size for increased verb retrieval in a sentence context following a-tDCS over Broca’s area compared to sham stimulation. Additionally, the observed effect of a-tDCS does not appear to be dependent on the engagement of attentional processes or mood levels. These findings suggest that tDCS may be a promising technique, when paired with speech-language treatment, for beneficially modulating lexical-retrieval outcomes in individuals with fluent aphasia.

Several studies, employing different tDCS paradigms, have shown that active stimulation alongside intensive behavioral language therapy is beneficial for production of trained nouns and/or verbs (Fiori et al., 2011, 2013; Kang et al., 2011; Marangolo et al., 2013; Costa et al., 2015). An important consideration, however, when working with clinical populations such as individuals with post-stroke aphasia is whether the intervention translates to improvements on items not targeted in therapy. Several studies examining the effect of tDCS on individuals with aphasia have not assessed generalization effects following treatment or have assessed generalization without an effect noted (e.g., Fiori et al., 2011, 2013; Marangolo et al., 2013; Vestito et al., 2014; Rosso et al., 2014; Costa et al., 2015). Our finding of generalized improvement for verbs is in line with Manenti et al. (2015), the only study that trained verbs and also found a generalization effect for verb treatment combined with tDCS.

One possible explanation as to why we observed a lexical retrieval benefit for untrained verbs in a sentence context may relate to the nature of the behavioral intervention implemented and combined with tDCS. We trained and tested verbs in the sentence context. Since verbs are rarely experienced in the single-word context, a sentence-level treatment approach likely has more real-world application than training at the single-word level even though there are examples of written signs in the environment where verbs (and nouns) are experienced in isolation (e.g., verbs: *enter, walk, stop*; e.g., nouns: *restroom*, *restaurant*, *parking-garage*).

Another aspect of the training program that may potentially have impacted performance was the inclusion of both semantic and phonological cues in all treatment tasks. The literature in the area of behavioral treatment for aphasia indicates intervention approaches that include both semantic and phonological cueing are superior to intervention approaches that implement one of these approaches alone (see Brady et al., 2012). In this case study all three forms of the behavioral treatment included both semantic and phonological cues, while focusing on sentence and discourse production as well. For example, in the modified SPPA (Helm-Estabrooks and Nicholas, 2000), the subject was provided with a picture prompt as well as a narrative scenario that included items semantically related to the target. In addition, the target sentence was modeled, and the subject was asked to repeat the model, which is a phonological cue. Therefore both semantic and phonological cueing were provided to promote noun and verb retrieval at the sentence level. As well, in the intervention that directly trained nouns and verbs in the sentence context, SEPT (modified from Raymer and Kohen, 2006), there was a picture prompt (a semantic cue), and repetition (a phonological cue), as well as a fill-in-the blank component (a semantic cue). Lastly the focused-discourse treatment involved both semantic and phonological cueing throughout and the clinician used both of these types of cues to promote successful communication and discussion about a topic generated from a newspaper article discussion.

With regard to our finding that a-tDCS exerted a significant benefit on verb retrieval and a moderate-to-strong effect size as compared to no significance for noun retrieval, at the sentence level, the literature indicates that frontal brain regions are implicated in verb naming, while both frontal and temporal areas underlie object naming (Lubrano et al., 2014)– though this view has been disputed (Crepaldi et al., 2011). By extension, our analyses also showed that there was no effect of a-tDCS on noun retrieval in isolation, which may be related to the non-invasive brain stimulation target; however, an additional explanation may have to do with the nature of the treatment as stated above (which targeted nouns and verbs in a sentence context and not in isolation). In other words, generalization may be less likely to occur in situations that differ from those experienced in the treatment sessions (Thompson, 1989). If noun and verb processing involves partially segregated neural structures, then the application of tDCS over Broca’s area alone in our study may account for why verb retrieval was enhanced and not noun retrieval to the same extent. This hypothesis is in accordance with a recent study that found that intensive language therapy coupled with a-tDCS (1 mA/20 min) over Broca’s area improves verb-naming accuracy in a group of chronic aphasics (Marangolo et al., 2013). Moreover, Manenti et al. (2015) found a similar effect alongside targeted speech treatment when a-tDCS was applied over the DLPFC (2 mA/20 min).

### Implications for Clinical Applications

Implementing non-invasive brain stimulation in conjunction with behavioral treatment of aphasia is a new research area in the field of aphasia rehabilitation. While initial studies have focused on translational application of tDCS in conjunction with word retrieval treatment approaches for single words, more recent approaches have included sentence-level treatments that have more ecological validity and may foster improved language production in closer to real-life communicative contexts than training of single words alone. Studies that demonstrate using tDCS as an adjuvant to behavioral treatment has an impact on generalization to untrained forms will certainly impact clinical approaches to aphasia rehabilitation in the future.

### Future Research

Translational application of tDCS for aphasia that combines a behavioral intervention with a brain stimulation technique is an exciting new research approach under investigation to promote aphasia rehabilitation. It is an obvious next step after the initial animal studies, and the proof-of-concept studies with the application of tDCS to the healthy adult population. In order to build on lessons learned from the behavioral intervention literature, however, future research that combines neuromodulation and behavioral intervention should include efficacious speech-language treatments that have real-world application and that have been shown to generalize to untrained forms even in the sham condition. That way, future studies can focus on the effect of the translational application of the tDCS independent of the behavioral therapy protocol, which will allow researchers to better evaluate the efficacy of tDCS.

### Limitations

Although the study provides evidence that including a combined behavioral treatment approach that involves training naming at the sentence-level with tDCS may increase sentence production, this case study is not without its limitations. Due to the use of a single-case design, the results need to be verified in a larger group of participants and can not be generalized to other people with aphasia until a larger group is studied. There is a major limitation on what can be learned from a single subject, since the typical variables to influence outcome such as age, gender, lesion size, and location do not play a role in single case studies. With regard to the targeted speech-language intervention, while training naming in the sentence context was implemented to promote an ecologically-focused treatment approach, there are limitations to how this method was employed as well as limitations with the outcome measures. Firstly, the focused discourse treatment was open-ended and the amount of cueing provided by the clinician was not controlled. Secondly, the sentence production probes used as outcome measures were balanced for frequency of nouns only; however, frequency of verbs as well as instrumentality of verbs should have been considered in these lists as well (Jonkers and Bastiaanse, 2007). In addition, while outcome measures in this study included an assessment of untrained nouns and verbs in the sentence context, outcome measures that assess whether communication experience gains following stimulation and speech treatment are functionally relevant, such as discourse measures, should be included to consider generalization to language experiences in the real world. As well, outcomes should be measured over time to determine the maintenance of any treatment effects. Finally, while the result that there was no improvement in the sham condition and improvement in the a-tDCS condition is cautiously interpreted as a positive result, finding an effect in the sham condition with a greater effect size in the a-tDCS condition would provide better evidence that our behavioral treatment is indeed efficacious. While implementing a behavioral treatment approach that involves training at the sentence and discourse levels rather than the single-word level was motivated by our clinical viewpoint that language and communication occur at a level higher than single words, we do not have research evidence that people with aphasia benefit from our behavioral treatment approach. A behavioral treatment approach such as the verb-argument structure treatment described by Thompson et al. (2013), which has been shown to demonstrate generalized treatment effects in behavioral therapy alone would more strongly support the notion that training at the sentence level is efficacious and generalizes to untrained productions.

## Conclusion

Our findings are consistent with prior research that has found a beneficial effect of a-tDCS applied over Broca’s area on verb retrieval. In our study this was observed in an individual with fluent, anomic aphasia who participated in word-retrieval therapy directed at the sentence level. Our results suggest that the translational use of tDCS as an adjuvant therapy in individuals with aphasia is feasible and may be efficacious. Further research combining tDCS and a behavioral treatment that has been documented to promote language improvement will further inform aphasia researchers regarding the efficacy of using tDCS as an adjuvant to behavioral intervention for aphasia.

## Conflict of Interest Statement

The authors declare that the research was conducted in the absence of any commercial or financial relationships that could be construed as a potential conflict of interest.
